# Gracilis Muscle Flap Reconstruction for Refractory Urethral Complications After Pelvic Surgery: A Report of Two Cases

**DOI:** 10.1002/iju5.70283

**Published:** 2026-10-14

**Authors:** Akihiro Maeda, Shohei Tobu, Minika Yukimoto, Shuhei Kusano, Maki Kawasaki, Hiroaki Kakinoki, Mitsuru Noguchi

**Affiliations:** ^1^ Department of Urology, Faculty of Medicine Saga University Saga Japan

**Keywords:** prostatectomy, reconstructive surgical procedures, rectal fistula, robotic surgical procedures, surgical flaps

## Abstract

**Introduction:**

Iatrogenic urethral injury and rectourethral fistula are rare and challenging complications of pelvic surgery.

**Case Presentation:**

Case 1 involved a 72‐year‐old man with persistent urinary leakage after primary repair of a prostatic urethral injury sustained during rectal cancer surgery following neoadjuvant chemoradiotherapy. Approximately 2 years later, reclosure of the urethral defect with local periurethral reinforcement and gracilis muscle flap coverage enabled catheter removal and spontaneous voiding. Case 2 involved a 62‐year‐old man who developed a rectourethral fistula after robot‐assisted radical prostatectomy. After conservative management failed, fistulectomy, rectal and urinary tract repair, and gracilis muscle flap interposition achieved fistula closure and subsequent colostomy reversal.

**Conclusion:**

These cases illustrate two distinct reconstructive roles of the gracilis muscle flap: vascularized reinforcement of a compromised urethral repair and interposition between repaired urinary and rectal sites.

## Keynote Message

Gracilis muscle flaps can serve different reconstructive roles in refractory urethral complications after pelvic surgery. In Case 1, the flap provided vascularized reinforcement after secure reclosure of a chronic urethral defect, whereas in Case 2, it separated the repaired urinary and rectal sites. Secure reconstruction of the urinary tract remains essential, with the gracilis muscle flap serving as an adjunct.

## 1 Introduction

Iatrogenic urethral injuries and rectourethral fistulas are rare complications of pelvic surgery that may not heal with conservative treatment or primary closure [1, 2, 3, 4, 5, 6, 7]. Gracilis muscle flaps provide well‐vascularized tissue for refractory cases [7, 8, 9, 10, 11, 12]. We report two cases illustrating distinct reconstructive roles of this flap after pelvic surgery.

## 2 Case Presentation

### 2.1 Case 1

A 72‐year‐old man with cT3N3M0 rectal cancer underwent long‐course chemoradiotherapy consisting of pelvic irradiation (45 Gy) with capecitabine (2400 mg/day), followed by two cycles of capecitabine plus oxaliplatin and robot‐assisted abdominoperineal resection. During transperineal dissection, the urethral catheter was exposed through the prostatic urethra, confirming urethral injury. The urethral mucosa was repaired with a running 4‐0 absorbable suture, the surrounding tissue was approximated with 3‐0 absorbable sutures, and the repair site was covered with the peritoneum.

Postoperative urethrography revealed persistent contrast extravasation. As the patient prioritized rehabilitation for lower‐extremity dysfunction, definitive reconstruction was deferred. Extravasation was evident 18 months after the surgery (Figure 1A). Cystoscopy revealed a fistulous opening at the 7‐o'clock position relative to the verumontanum (Figure 1B).

**FIGURE 1:**
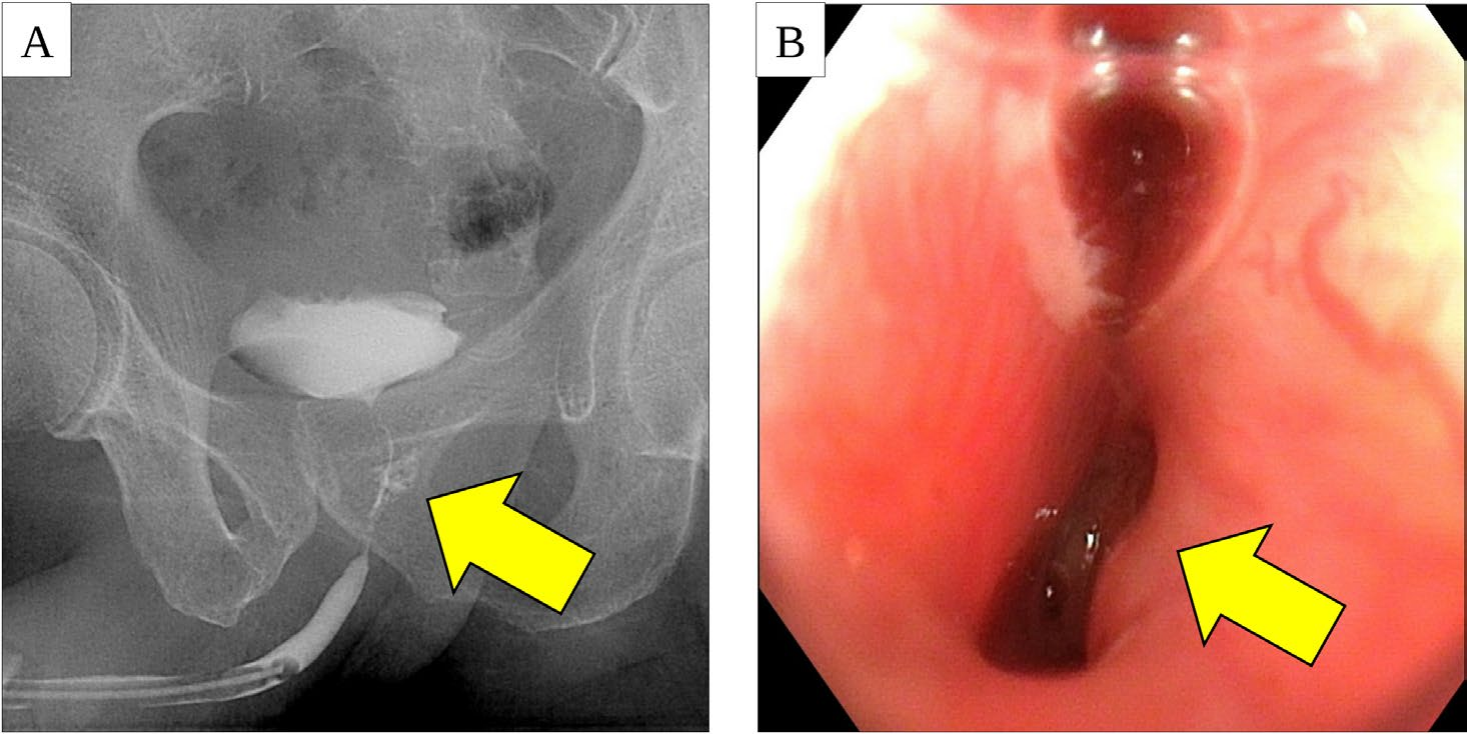
Preoperative findings in Case 1. (A) Urethrography shows contrast extravasation from the site of the initial urethral repair (arrow). (B) Cystoscopy shows a fistulous opening at the 7‐o'clock position relative to the verumontanum (arrow).

Approximately 2 years after the initial surgery, transperineal urethral repair was performed in collaboration with a plastic surgeon. The patient was placed in the prone split‐leg position. Because the rectum had already been resected, no posterior rectal landmark was available. The urethra was exposed and encircled through the perineal approach, and a flexible cystoscope was inserted through the external urethral meatus. Transillumination from the cystoscope was used to identify the urethral defect within the scarred operative field. The dissection was confined to the perineal operative field, and the peritoneal cavity was not entered. After trimming the fistulous opening and surrounding scar tissue, a urethral defect measuring slightly < 10 mm remained. The defect was closed with five interrupted 4‐0 polydioxanone sutures. The surrounding periurethral tissue was then mobilized as a local flap and secured over the urethral closure with interrupted 2‐0 polyglycolic acid sutures. A leak test confirmed a watertight closure. A pedicled gracilis muscle flap harvested from the right thigh was subsequently transposed to the perineum through a subcutaneous tunnel and placed over the reinforced urethral repair site (Figure 2). The operative time was 6 h 3 min, and the estimated blood loss was 70 mL.

**FIGURE 2:**
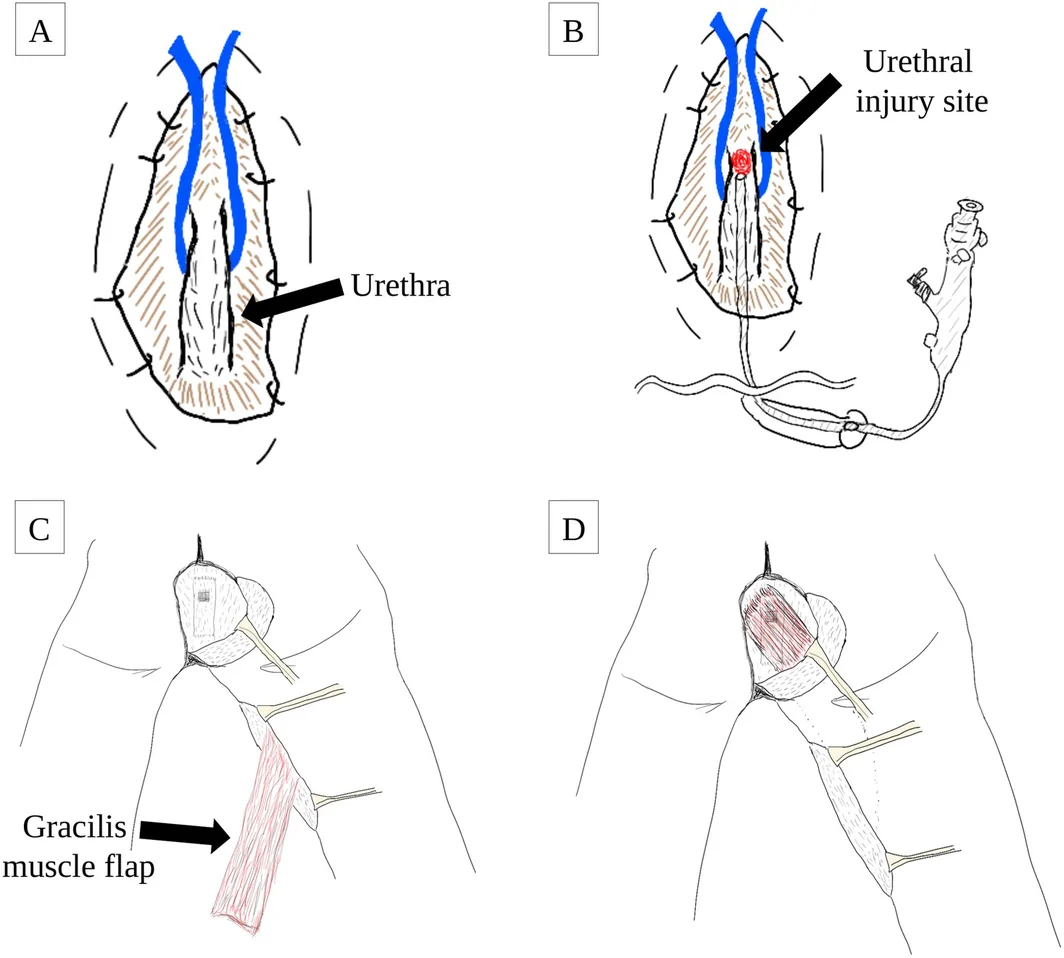
Schematic representation of urethral repair using a gracilis muscle flap in Case 1. (A) With the patient in the prone split‐leg position, the urethra was exposed and encircled through a perineal approach (arrow, urethra). (B) The site of urethral injury was identified using light from a flexible cystoscope inserted through the external urethral meatus (arrow, urethral injury). (C) A pedicled gracilis muscle flap was harvested from the right thigh (arrow, gracilis muscle flap). (D) The flap was transposed to the perineum through a subcutaneous tunnel and placed over the urethral repair site.

Urethrography performed 2 months postoperatively showed no extravasation, and the urethral catheter was removed. At 7 months after repair, uroflowmetry showed a voided volume of 222 mL, maximum flow rate of 16.0 mL/s, and post‐void residual volume of 33 mL. The patient continued to void spontaneously without wound dehiscence or gait disturbances.

### 2.2 Case 2

A 62‐year‐old man with cT3aN0M0 prostate cancer underwent robot‐assisted radical prostatectomy after 1 year of neoadjuvant combined androgen blockade. Although no obvious rectal injury was identified intraoperatively, pneumaturia developed on Postoperative Day 14, and cystoscopy revealed a rectourethral fistula (Figure 3A). A urethral catheter was placed, and a diverting sigmoid colostomy was created on Postoperative Day 24.

**FIGURE 3:**
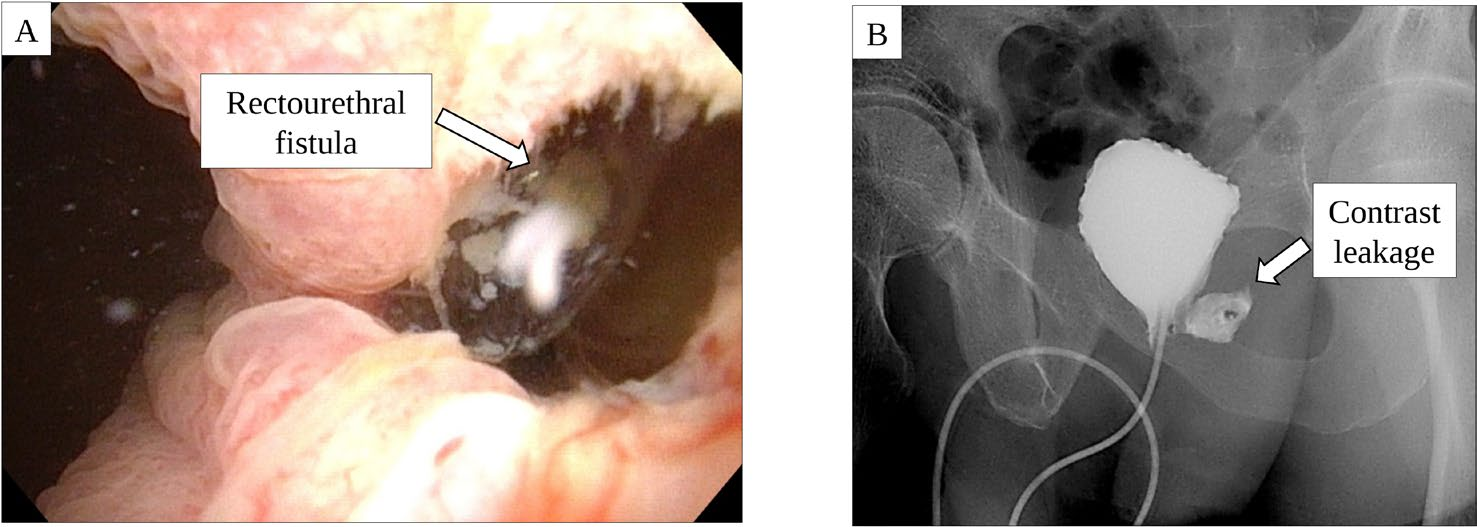
Diagnostic findings of the rectourethral fistula in Case 2. (A) Cystoscopy shows the opening of the rectourethral fistula (arrow). (B) Urethrography shows contrast extravasation through the fistula (arrow).

Three months later, urethrography continued to show contrast extravasation through the fistula (Figure 3B). Six months after radical prostatectomy, transperineal repair was performed in collaboration with colorectal and plastic surgeons. The patient was placed in the lithotomy position. Through a perineal approach, the fistulous tract was dissected and excised by the colorectal surgeons, followed by repair of the rectal defect. After fistula excision, the vesicourethral anastomosis remained open. The bladder and urethral mucosa were reapproximated with a running 4‐0 polydioxanone suture, followed by additional interrupted 4‐0 polydioxanone sutures to approximate the outer bladder and urethral layers. A flexible cystoscope was inserted through the external urethral meatus, confirming satisfactory mucosal approximation between the bladder and urethra. Irrigation through the cystoscope showed no leakage into the operative field. A pedicled gracilis muscle flap harvested from the left thigh was then interposed between the repaired vesicourethral anastomosis and rectal repair site (Figure 4A). The operative time was 8 h 20 min, and the estimated blood loss was 125 mL.

**FIGURE 4:**
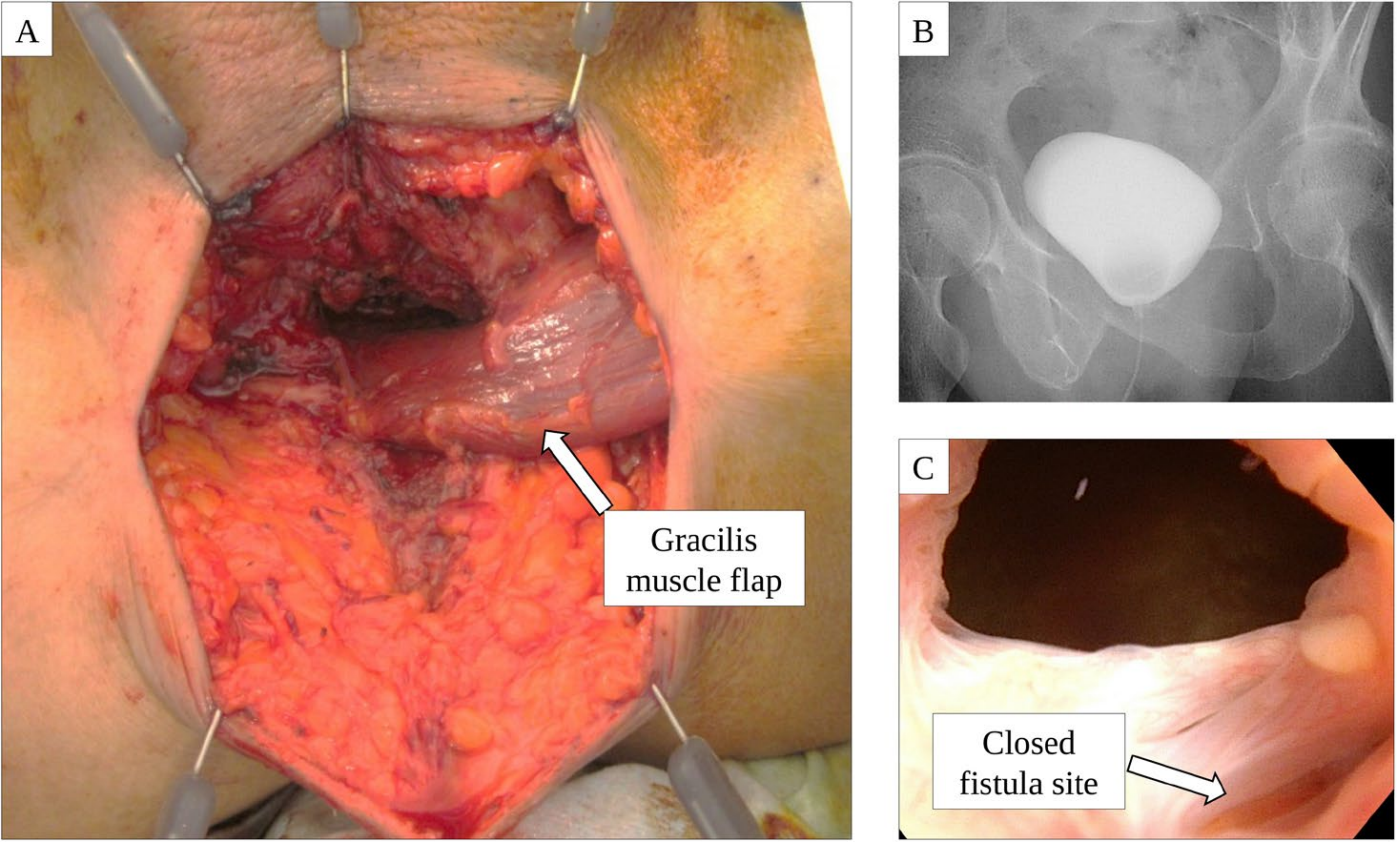
Findings during and after rectourethral fistula repair using a gracilis muscle flap in Case 2. (A) After fistulectomy and rectal repair, the gracilis muscle flap was interposed between the repaired vesicourethral anastomosis and the rectal repair site. (B) Cystography on Postoperative Day 16 shows no contrast extravasation. (C) Cystoscopy at 5 months after repair confirms fistula closure (arrow, closed fistula site).

Cystography performed on Postoperative Day 16 showed no extravasation, and the urethral catheter was removed (Figure 4B). Cystoscopy performed at 5 months confirmed fistula closure (Figure 4C). The colostomy was reversed 2 months after repair. At the final follow‐up, 46 months after the repair, no fistula recurrence was observed and satisfactory urinary continence was maintained.

## 3 Discussion

Urethral injury during rectal cancer surgery and rectourethral fistula after minimally invasive radical prostatectomy are rare, occurring in 1.64% and 0.09% of patients, respectively [1, 2]. Primary suture repair is standard for intraoperatively recognized urethral injury. However, repair may fail, particularly in compromised tissues [6, 13]. In Case 1, preoperative radiotherapy may have impaired tissue perfusion and wound healing.

Rectourethral fistula treatment should be individualized according to fistula size, radiotherapy history, urethral stricture, and tissue condition [14]. Transperineal repair with well‐vascularized tissue is useful for complex or persistent fistulas [7, 8, 10, 11, 15]. The gracilis muscle flap can reinforce repairs, obliterate dead space, provide vascularized tissue, and separate urinary and rectal suture lines, with generally limited effect on lower‐extremity function [16, 17].

Importantly, the gracilis flap is an adjunct rather than a substitute for secure urinary tract closure. Adequate debridement and tension‐free, watertight closure are prerequisites; in both cases, absence of leakage was confirmed before flap placement. This approach is technically demanding and entails an additional donor‐site procedure, multidisciplinary surgical expertise, and prolonged operative time. Operative times were 6 h 3 min and 8 h 20 min in Cases 1 and 2, which represent a potential drawback of this multidisciplinary approach.

Previous reports have mainly described gracilis muscle flap interposition for rectourethral fistula repair [7, 8, 9, 10, 11]. Gracilis coverage of a tubularized buccal mucosal graft has also been reported for posterior urethral reconstruction after abdominoperineal resection [18]. In contrast, Case 1 used the flap as vascularized reinforcement after secure reclosure of the native urethra without substitution, whereas Case 2 used it as an interposition barrier between repaired urinary and rectal sites. These cases therefore demonstrate two distinct reconstructive roles: reinforcement of a compromised urethral repair and separation of urinary and gastrointestinal suture lines. The gracilis flap may thus be tailored to the reconstructive problem encountered during salvage surgery. Additional cases are needed to evaluate these applications and long‐term outcomes.

## 4 Conclusion

These cases illustrate that a gracilis muscle flap can serve distinct adjunctive roles as vascularized reinforcement of a compromised urethral repair or as interposition between repaired urinary and rectal sites.

## Funding

The authors have nothing to report.

## Ethics Statement

The authors have nothing to report.

## Consent

Written informed consent for the publication of this case report and the accompanying images was obtained from both patients.

## Conflicts of Interest

The authors declare no conflicts of interest.

## Data Availability

The data supporting the findings of this case report are available from the corresponding author upon reasonable request, subject to patient privacy and ethical restrictions.

## References

[1] P. Planellas , L. Cornejo , A. Ehsan , et al., “Urethral Injury in Rectal Cancer Surgery: A Comprehensive Study Using Cadaveric Dissection, Imaging Analyses, and Clinical Series,” Cancers (Basel) 15, no. 20 (2023): 4955. 10.3390/cancers15204955 PMC10605354 37894322

[2] F. Rabbani , L. H. Yunis , R. Pinochet , et al., “Comprehensive Standardized Report of Complications of Retropubic and Laparoscopic Radical Prostatectomy,” European Urology 57, no. 3 (2010): 371–386. 10.1016/j.eururo.2009.11.034 19945779

[3] Japanese Urological Association , Clinical Practice Guideline for Urological Trauma, 2022 Edition [in Japanese] (Igaku Tosho Shuppan, 2022).

[4] European Association of Urology , “EAU Guidelines on Urological Trauma,” Presented at the EAU Annual Congress, London 2026 (EAU Guidelines Office, 2026).

[5] A. R. Mundy and D. E. Andrich , “Urorectal Fistulae Following the Treatment of Prostate Cancer,” BJU International 107, no. 8 (2011): 1298–1303. 10.1111/j.1464-410X.2010.09686.x 20883482

[6] P. Sylla , J. J. Knol , A. P. D'Andrea , et al., “Urethral Injury and Other Urologic Injuries During Transanal Total Mesorectal Excision: An International Collaborative Study,” Annals of Surgery 274, no. 2 (2021): e115–e125. 10.1097/SLA.0000000000003597 31567502

[7] M. Lo Re , M. Pezzoli , E. Garcia Rojo , et al., “A Systematic Review on the Surgical Management of Acquired Rectourethral Fistula,” International Journal of Impotence Research 38, no. 3 (2026): 214–225. 10.1038/s41443-025-01100-y 40579441

[8] O. Zmora , F. M. Potenti , S. D. Wexner , et al., “Gracilis Muscle Transposition for Iatrogenic Rectourethral Fistula,” Annals of Surgery 237, no. 4 (2003): 483–487. 10.1097/01.SLA.0000059970.82125.DB PMC1514481 12677143

[9] T. Takeda , T. Shonaka , C. Tani , T. Hayashi , H. Kakizaki , and Y. Sumi , “Gracilis Muscle Flap Combined With a Laparoscopic Transabdominal Approach Is Effective in the Treatment of Post‐Prostatectomy Rectourethral Fistula: A Case Report,” International Journal of Surgery Case Reports 92 (2022): 106856. 10.1016/j.ijscr.2022.106856 PMC8917308 35276434

[10] M. Sbizzera , N. Morel‐Journel , A. Ruffion , et al., “Rectourethral Fistula Induced by Localised Prostate Cancer Treatment: Surgical and Functional Outcomes of Transperineal Repair With Gracilis Muscle Flap Interposition,” European Urology 81, no. 3 (2022): 305–312. 10.1016/j.eururo.2021.09.017 34686386

[11] G. Yankelevich , J. Swaim , I. Coate , et al., “Post‐Operative Outcomes in Rectourethral Fistula Repair Using Gracilis Interposition Flap,” Canadian Journal of Urology 32, no. 1 (2025): 37–42. 10.32604/cju.2025.064684 40194934

[12] N. Wada , H. Takagi , T. Ishimaru , et al., “A Case of Achieving Urinary Continence Using Gracilis Muscle Flap for Recanalization and Fistula After Bladder Neck Closure,” IJU Case Reports 8, no. 3 (2025): 227–230. 10.1002/iju5.70012 PMC12055205 40336736

[13] S. Kimura , S. Niikura , and Y. Ishiura , “Immediate Urethral Reconstruction for Membranous Urethral Injury During Rectal Surgery: A Case Report,” IJU Case Reports 9, no. 3 (2026): e70188. 10.1002/iju5.70188 PMC13168528 42137724

[14] M. Poitevin , M. Ferragu , P. Bigot , T. Culty , and A. Venara , “Rectourethral Fistulas After Treatment for Prostate Carcinoma: Update and New Management Algorithm,” Journal of Visceral Surgery 162, no. 3 (2025): 199–208. 10.1016/j.jviscsurg.2025.01.010 39952891

[15] S. H. Van Der Graaf , E. M. K. Wit , G. L. Beets , et al., “Management in Robot‐Assisted Radical Prostatectomy Patients With Recto‐Urethral Fistulas: The York–Mason Technique,” World Journal of Urology 43, no. 1 (2025): 604. 10.1007/s00345-025-05996-5 PMC12515200 41074986

[16] M. Juricic , P. Vaysse , J. Guitard , J. Moscovici , J. Becue , and S. Juskiewenski , “Anatomic Basis for Use of a Gracilis Muscle Flap,” Surgical and Radiologic Anatomy 15, no. 3 (1993): 163–168. 10.1007/BF01627695 8235956

[17] M. E. Lyons and J. J. Goldman , “Gracilis Tissue Transfer,” in StatPearls [Internet] (StatPearls Publishing, 2026). 32644472

[18] S. V. Parab , G. Goyal , S. Jadhav , H. Pathak , M. Andankar , and C. Gadekar , “The Use of Gracilis Muscle Flap as a Cover of Tabularized Buccal Mucosal Graft in the Posterior Urethral Injury,” Urology Annals 12, no. 2 (2020): 176–179. 10.4103/UA.UA_9_19 PMC7292441 32565658

